# Does an increase in body mass index over 10 years affect knee structure in a population-based cohort study of adult women?

**DOI:** 10.1186/ar3078

**Published:** 2010-07-13

**Authors:** Sharon L Brennan, Flavia M Cicuttini, Julie A Pasco, Margaret J Henry, Yuanyuan Wang, Mark A Kotowicz, Geoff C Nicholson, Anita E Wluka

**Affiliations:** 1 School of Public Health and Preventive Medicine, Department of Epidemiology and Preventive Medicine: Monash University, Commercial Road, Melbourne 3004, Australia; 2 Department of Clinical and Biomedical Sciences: Barwon Health, The University of Melbourne, Ryrie Street, Geelong 3220, Australia

## Abstract

**Introduction:**

Although obesity is a modifiable risk factor for knee osteoarthritis (OA), the effect of weight gain on knee structure in young and healthy adults has not been examined. The aim of this study was to examine the relationship between body mass index (BMI), and change in BMI over the preceding 10-year period, and knee structure (cartilage defects, cartilage volume and bone marrow lesions (BMLs)) in a population-based sample of young to middle-aged females.

**Methods:**

One hundred and forty-two healthy, asymptomatic females (range 30 to 49 years) in the Barwon region of Australia, underwent magnetic resonance imaging (MRI) during 2006 to 2008. BMI measured 10 years prior (1994 to 1997), current BMI and change in BMI (accounting for baseline BMI) over this period, was assessed for an association with cartilage defects and volume, and BMLs.

**Results:**

After adjusting for age and tibial plateau area, the risk of BMLs was associated with every increase in one-unit of baseline BMI (OR 1.14 (95% CI 1.03 to 1.26) *P *= 0.009), current BMI (OR 1.13 (95% CI 1.04 to 1.23) *P *= 0.005), and per one unit increase in BMI (OR 1.14 (95% CI 1.03 to 1.26) *P *= 0.01). There was a trend for a one-unit increase in current BMI to be associated with increased risk of cartilage defects (OR 1.06 (95% CI 1.00 to 1.13) *P *= 0.05), and a suggestion that a one-unit increase in BMI over 10 years may be associated with reduced cartilage volume (-17.8 ml (95% CI -39.4 to 3.9] *P *= 0.10). Results remained similar after excluding those with osteophytes.

**Conclusions:**

This study provides longitudinal evidence for the importance of avoiding weight gain in women during early to middle adulthood as this is associated with increased risk of BMLs, and trend toward increased tibiofemoral cartilage defects. These changes have been shown to precede increased cartilage loss. Longitudinal studies will show whether avoiding weight gain in early adulthood may play an important role in diminishing the risk of knee OA.

## Introduction

Obesity is recognised as a modifiable risk factor for knee OA, and increased body mass index (BMI) is consistently associated with the risk of large joint OA [1-4]. In the elderly, weight loss of as little as two BMI units over 12 years has been shown to reduce the risk of knee OA [5]. However, the age at which weight gain in early adulthood begins to impact on knee structure and increase the risk of knee OA is unknown [6]. In middle-aged asymptomatic adults, obesity has been associated with increased prevalence of cartilage defects, the earliest structural change of OA [2,7] that is associated with cartilage loss, radiographic severity of OA, and is an independent predictor of knee joint replacement [8]. It is important to study these relationships in younger age groups since the mechanical properties of joint structures, such as cartilage, differ with ageing [9]. However the effect of obesity and weight gain on knee structure in younger adults with no radiographic knee OA, and whether this affects longitudinal structural changes has not been examined.

Osteoarthritis (OA) is a disease of the whole joint, characterised by a number of structural changes including the development of cartilage defects and reduction in the amount of articular cartilage, bone marrow lesions (BMLs) and metaphyseal expansion [10]. Although these changes are more pronounced in those with established OA, structural changes are also present prior to the clinical and radiographic presentation of OA, in preclinical and pre-radiographic disease [11]. By the time the first signs of radiographic disease are present, even with grade 1 joint space narrowing, 10% of articular cartilage loss has already occurred [11]. Use of MRI enables the examination of knee structure on a continuum from the normal knee to one with OA, enabling preclinical and pre-radiographic disease to be examined. Various measures of cartilage can be quantified, and reflect different dimensions of the pathophysiological process. For example, knee cartilage volume has been shown to correlate with radiological OA [11,12] and predicts joint replacement. Complementary information is obtained by identifying cartilage defects that, independent of cartilage volume, are associated with subsequent cartilage volume loss in asymptomatic subjects with no radiological OA [13,14], and also predict cartilage loss and joint replacement in those with knee OA [15]. Furthermore, other structural abnormalities such as bone marrow lesions (BMLs), which are associated with knee pain and predict cartilage damage, can also be examined [16,17].

The aim of this study was to determine the relationship between baseline BMI, current BMI, and change in BMI over 10 years with knee structure (cartilage volume, defects, and BMLs), in healthy, population-based young to middle-aged females without radiographical or clinical knee OA.

## Materials and methods

### Subjects

Data were derived from a population-based, age-stratified, random sample of 1,494 adult females enrolled in the Geelong Osteoporosis Study (GOS), recruited from Commonwealth electoral rolls for the Barwon Statistical Division (BSD), Australia, during 1994 to 1997 [18]. Of these, 1,071 attended the 10-year follow up during 2004 to 2007 (71.7% retention), and 352 women were eligible for this study based on initial inclusion criteria of age range 30 to 49 years, and still resident within the BSD. Of these, 140 women (39.8%) could not be contacted, and 41 (11.6%) declined. Potential participants were excluded if any of the following were present: knee OA as described by the American College of Rheumatology clinical criteria [19]; knee pain lasting >24 hours during the previous five years (*n *= 1); previous knee injury requiring non-weight bearing treatment >24 hours, or surgery (including arthroscopy) (*n *= 18); a history of any form of arthritis as diagnosed by a medical practitioner (*n *= 2); contraindication to MRI including pregnancy (*n *= 2), pacemaker, metal sutures (*n *= 1), presence of shrapnel or iron filings in the eye, or claustrophobia (*n *= 5). One hundred and forty-two women (40.3%) were thus eligible to participate in this study. Radiographs were not performed. All participants provided informed written consent. Approval for the study was obtained from the Barwon Health Human Research Ethics Committee and Monash University Human Research Ethics Committee.

### Anthropomorphic measures

Weight and height were measured at baseline (1994 to 1997) and current (2004 to 2007) to the nearest ±0.1 kg and ±0.1 cm, respectively. BMI was calculated as weight/height squared (kg/m^2^) at baseline and current at 10-year follow up. Change in BMI over 10 years was calculated.

### MRI

An MRI was performed at the 10-year follow-up on the dominant knee of each subject, defined as the self-selected lower limb which the subject used to kick a ball. Knees were imaged at Barwon Medical Imaging in the sagittal plane on a 1.5-T whole body magnetic resonance unit (Philips, Eindhoven, the Netherlands) using a commercial transmit-receive extremity coil. The following parameters and sequences were applied: a T_1_-weighted fat suppressed 3D gradient recall acquisition in the steady state; flip angle 55 degrees; repetition time 58 msec; echo time 12 msec; field of view 16 cm; 60 partitions; 512 × 512 matrix; one acquisition time 11 minutes 56 seconds. Sagittal images were obtained at a partition thickness of 1.5 mm and an in-plane resolution of 0.31 × 0.31 mm (512 × 512 pixels). In addition, a T_2_-weighted coronal fat-saturated acquisition, repetition time 2,200 ms, echo time 20/80 ms, with a slice thickness of 3 mm, a 0.3 interslice gap, one excitation, a field of view of 11 to 12 cm, and a matrix of 256 × 256 pixels was also obtained [20].

### The assessment of cartilage defects

Cartilage defects in the medial and lateral tibial femoral cartilages were graded on the MR images with a classification system as previously described [14,21]. A cartilage defect was identified as present if there was irregularity on the cartilage surface with loss of cartilage thickness on at least two consecutive slices. Once ascertained, tibial (*n *= 51) and femoral (*n *= 55) cartilage defects were combined to measure prevalence of tibiofemoral defects. Intraobserver reliability and interobserver reliability have previously been assessed in 50 MR images (expressed as intraclass correlation coefficient, ICC), and found to be 0.90 and 0.90 for the medial tibiofemoral compartment, and 0.89 and 0.85 for the lateral tibiofemoral compartment, respectively [21].

### The assessment of cartilage volume

Tibial cartilage volume (ml) at the medial and lateral compartments was determined from the MRI images using Osiris software (Geneva, Switzerland), as previously described [22]. These were summed to create total tibial cartilage volume. The coefficient of variation (CV) for cartilage volume measures have been reported as 2.1% for the medial tibial and 2.2% for lateral tibial cartilage [23].

### The measurement of tibial plateau area

Medial and lateral cross-sectional areas of tibial plateau bone were determined by creating an isotropic volume from the input images that were reformatted in the axial plane. Areas were directly measured from these images. Using this technique, osteophytes, if present, are not included in the area of interest [24]. A single, trained reader measured all tibial plateau areas, which were unpaired, and blinded to both subject identification and time sequence. Medial and lateral tibial plateau bone area was summed to obtain tibial plateau bone area. CV have been assessed for the medial and lateral tibial plateau, and found to be 2.3% and 2.4%, respectively [23,25].

### The assessment of BMLs

BMLs were defined as areas of increased signal intensity adjacent to subcortical bone in either the medial or lateral distal femur or the proximal tibia [26]. Two trained, blinded observers, assessed the presence or absence of lesions for each subject [17]. A lesion was defined as present if it appeared on at least two or more adjacent slices and encompassed at least one quarter of the width of the tibial or femoral cartilage being examined from coronal images, comparable to at least a *grade 2 *BML described by Felson *et al *[17]. The reproducibility for determination of BMLs was assessed using 60 randomly selected knee MRIs and found to have high agreement (κ value 0.88, *P *< 0.001) [27,28].

### The assessment of osteophytes

The presence of osteophytes was determined from the T_2_-weighted coronal fat-saturated acquisition images. Use of MRI to detect osteophytes has been shown to be more sensitive than X-rays [29]. Osteophytes were measured from coronal images by two independent trained observers. In the event of disagreement between observers, a third independent observer reviewed the MRI. Intra-observer and inter-observer reproducibility for agreement on osteophytes (yes/no) ranged between 0.85 and 0.93 (κ statistic).

### Statistical analysis

Binary logistic regression was used to assess the relationship between baseline, current, and every one unit change in BMI kg/m^2 ^over the 10-year period (the latter accounting for baseline BMI), with the presence of BMLs and cartilage defects, and multivariable linear regression for cartilage volume. Models were adjusted for age and tibial plateau area. Interaction terms were checked for effect modification. Analysis was performed on all the participants, and then in those without osteophytes, to examine the relationship in those highly unlikely to have radiographic OA, since the MRI is more sensitive in detecting osteophytes than radiographs [29]. Significance was set at *P *< 0.05 and statistical analyses were performed using MINITAB (Version 15.0; Minitab, State College, PA, USA) and SPSS (Version 15.0; SPSS, Cary, NC, USA).

## Results

The demographic characteristics of the total study population (*n *= 142) are presented in Table 1. Over the 10-year study period, mean measures of obesity increased (weight +6.14 ± 0.71 kg, BMI +2.45 ± 0.27 kg/m^2^, both *P *< 0.0001). At baseline, 88 (62.0%) had normal BMI (<25 kg/m^2^), 37 (26.1%) were overweight (25 to 29.9 kg/m^2^), and 17 (12.0%) were obese (≥30 kg/m^2^). Over the study period, the change in weight ranged from -11.5 kg to +37.3 kg. At the 10-year follow up, 62 (43.7%) had normal BMI, 42 (29.6%) were overweight, and 38 (26.8%) were obese. Current BMI of the study sample was 1.4 kg less than subjects not included for analysis (*P *< 0.001). Thirteen participants had osteophytes present.

**Table 1:** Characteristics of study population (*n *= 142)

Characteristic	Value
Age (yr)	41.7 ± 5.3^a^
Height (cm)	163.6 ± 5.8^a^
Weight (kg)	
baseline	66.9 ± 14.1^a^
current	73.0 ± 16.7^a^
change	4.6 (0.9 to 11.1)^b^
Body mass index (kg/m^2^)	
baseline	25.0 ± 5.0^a^
current	27.3 ± 6.3^a^
change	1.8 (0.3 to 4.2)^b^
Any significant tibiofemoral cartilage defect, number = (%)	76 (53.5%)^c^
Tibial cartilage volume (ml)	23.1 ± 3.9^a^
Tibial plateau bone area (cm^2^)	30.2 ± 2.4^a^
Presence of bone marrow lesions, number = (%)	9 (6.3%)^c^
Osteophytes, number = (%)	13 (9.2%)

Results presented as^a^mean ± SD,^b^median (IQR range), or^c^frequency (%).

We examined the relationship between baseline BMI, current BMI, and a one unit increase in change in BMI over the 10-year period (adjusting for baseline BMI) and current knee structures (Table 2). In univariate analyses no association was observed with cartilage volume (Table 2) (*P *= 0.2 to 0.9). After adjusting for age and tibial plateau area, there was a tendency for reduced cartilage volume to be associated with a one unit increase in BMI over the preceding 10 years in the total population (-17.8 ml (95% CI -39.4 to 3.9) *P *= 0.10). To ensure that these results were not due to early preclinical OA in our participants (that is, osteophytes present), we performed a subgroup analysis, excluding those with osteophytes, and obtained similar results (data not shown). Similar results were observed in the medial and lateral compartments (data not shown).

**Table 2:** Relationship between BMI (kg/m^2^), and cartilage properties and bone marrow lesions in the tibiofemoral compartment

	Univariate analysis *n *= 142	*P* value	**Multivariate analysis**^**a **^***n *= 142**	*P* value	**Multivariate analysis**^**b **^***n *= 129**	*P *value
*Cartilage volume (ml)*						
Current BMI	-4.20 (-14.58, 6.18)^1^	0.42	-6.98 (-16.98, 3.02)^2^	0.17	-7.73 (-18.55, 3.09)^2^	0.16
Baseline BMI	-0.60 (-13.51, 12.32)^1^	0.93	-3.81 (-16.65, 9.03)^2^	0.55	-5.04 (-19.41, 9.33)^2^	0.48
Change in BMI	-14.88 (-35.01, 5.26)^1^	0.15	-16.44 (-35.66, 2.78)^3^	0.09	-17.75 (-39.41, 3.91)^3^	0.10

*Tibiofemoral cartilage defects (yes/no)*						
Current BMI	1.08 (1.02, 1.15)^4^	**0.01**	1.06 (1.00, 1.13)^5^	0.050	1.07 (1.00, 1.14)^5^	0.050
Baseline BMI	1.09 (1.01, 1.18)^4^	**0.02**	1.06 (0.98, 1.15)^5^	0.13	1.05 (0.97, 1.14)^5^	0.25
Change in BMI	1.08 (0.97, 1.20)^4^	0.15	1.08 (0.96, 1.21)^6^	0.20	1.13 (1.00, 1.29)^6^	0.060

*Bone marrow lesions (yes/no)*						
Current BMI	1.13 (1.04, 1.23)^7^	**0.005**	1.13 (1.04, 1.23)^8^	**0.005**	1.14 (1.03, 1.27)^8^	**0.01**
Baseline BMI	1.14 (1.03, 1.26)^7^	**0.009**	1.14 (1.03, 1.26)^8^	**0.009**	1.17 (1.02, 1.33)^8^	**0.02**
Change in BMI	1.15 (0.96, 1.38)^7^	0.14	1.14 (1.03, 1.26)^9^	**0.01**	1.12 (0.88, 1.43)^9^	0.36

Total population of *n *= 142^a^, and subgroup analysis of those without osteophytes, *n *= 129^b^.

BMI, body mass index.

^1 ^Difference in cartilage volume per unit increase in BMI.

^2 ^Difference in cartilage volume per unit increase in BMI adjusted for age, tibial plateau area.

^3 ^Difference in cartilage volume per unit increase in BMI adjusted for age, tibial plateau area, and baseline BMI.

^4 ^Odds of tibiofemoral cartilage defects per unit increase in BMI.

^5 ^Odds of tibiofemoral cartilage defects per unit increase in BMI adjusted for age, tibial plateau area.

^6 ^Odds of tibiofemoral cartilage defects per unit increase in BMI adjusted for age, tibial plateau area, and baseline BMI.

^7 ^Odds of bone marrow lesions per unit increase in BMI.

^8 ^Odds of bone marrow lesions unit increase in BMI adjusted for age.

^9 ^Odds of tibiofemoral cartilage defects per unit increase in BMI adjusted for age and baseline BMI.

The presence of tibiofemoral cartilage defects was associated with current BMI (*P *= 0.01), and persisted after adjustment for age and tibial plateau area in the total population (OR 1.06 (95% CI 1.00 to 1.13) *P *= 0.05) and after excluding those with osteophytes (OR 1.07 (95% CI 1.00 to 1.14) *P *= 0.05, Table 2). Similar results were observed in the medial and lateral compartments (data not shown). After adjusting for the presence of BMLs, this relationship remained in the total population (OR 1.06 (95% CI 0.99 to 1.13) *P *= 0.09).

Greater BMI, both baseline and current, was associated with the presence of BMLs (yes/no) in logistic regression (OR 1.14 (95% CI 1.03 to 1.26) *P *= 0.009, OR 1.13 (95%vCI 1.04 to 1.23) *P *= 0.005, respectively, Table 2), and remained significant after adjusting for age (both *P *≤ 0.01). After adjusting for age, a one-unit increase in BMI over 10 years was associated with BMLs (OR 1.14 (95% CI 1.03 to 1.26) *P *= 0.01). After excluding those with osteophytes, only six participants with BML remained. After excluding participants with osteophytes, the age-adjusted results remained significant for an association between both baseline and current BMI, and BMLs (OR 1.14 (95% CI 1.03 to 1.27) *P *= 0.01, OR 1.17 (95% CI 1.02 to 1.33) *P *= 0.02, respectively) but change in BMI was not significantly associated with BMLs (OR 1.12 (95% CI 0.88 to 1.43) *P *= 0.36).

## Discussion

The findings of this study showed that in young to middle aged, healthy women without clinical OA, prior and current obesity, as well as increasing weight, is associated with detrimental changes to knee structure. Baseline and current BMI were associated with current BMLs. Even after adjusting for baseline BMI, further increase in BMI over the 10 years was independently associated with an increased risk of BMLs. Both current and increase in BMI (independent of baseline BMI) over 10 years showed a consistent pattern of association with the presence of tibiofemoral cartilage defects (*P *= 0.05 and *P *= 0.06 respectively).

It is known that those currently obese are at increased risk of BMLs [30]; however, we showed that an increased risk of BMLs was associated with increases in BMI, independent of baseline BMI. BMLs have an important role in knee OA, being associated with pain [17,28,31] and the adverse structural outcomes of increased joint space narrowing [16] and loss of cartilage volume [32,33]. Even in asymptomatic populations, such as within the current study, BMLs have also been associated with detrimental effects on cartilage, with increased prevalence of cartilage defects [30,34] and volume loss [32,33]. Although BMLs may be the result of trauma [35,36] or malalignment [16], there is also evidence that they may be affected by systemic factors [37,38]. Whether the mechanism for the association of obesity with BMLs is biomechanical or metabolic is unclear but there are data supporting both [39,40]. In either case, there is evidence that in asymptomatic populations, BMLs may resolve [28]; however, it is unknown whether weight loss may facilitate this. Because of the strong relationship between BMLs and subsequent cartilage loss, these data suggest that weight gain even in young adulthood is detrimental to knee structure and may increase the risk of OA.

Our data showed a consistent trend toward a detrimental effect of weight and weight gain on cartilage defects. Whilst the relationship between weight gain and cartilage volume did not achieve statistical significance, the direction of effect was similar. Cartilage defects occur early in the pathogenesis of OA, being present prior to clinical and radiographic disease, and prior to loss of cartilage volume. Whilst their presence is independent of cartilage volume, cartilage defects show a weak association with pain [41] and are predictors of increased cartilage loss, being an earlier stage of the disease process than loss of cartilage volume [14]. Thus, in this population-based, asymptomatic population we may be beginning to see an effect of BMI and change in BMI on cartilage defects, which occur at an earlier stage of disease than subsequent loss of cartilage volume. Whilst neither relationship achieved statistical significance, these relationships with defects were stronger when those with osteophytes were excluded from analysis. In addition, other factors such as malalignment which may mediate the effect of BMI on knee structure, as has been shown in OA [42], may play a role and warrants further study.

Cartilage defects are evidence of early cartilage pathology, independent of cartilage volume [21]. In asymptomatic subjects with no radiological OA, the presence of cartilage defects is associated with cartilage loss [14]. Thus, in a young to middle-aged, asymptomatic population, prevalent cartilage defects are more likely to be present than any reduction in cartilage volume, since these represent the early changes of OA, with a longer time frame required to demonstrate significant cartilage loss. It may be that this study did not have the power to detect an association between BMI and cartilage volume. However, we did demonstrate that even in younger asymptomatic adults obesity adversely affects knee structure, with particular effect on BMLs, but also with evidence of detrimental effects on cartilage, as evidenced by cartilage defects.

The strengths of this study are the objective measurement of obesity obtained 10 years prior to measurement of cartilage volume, defects and BMLs. This is the first study in asymptomatic, young to middle-aged females that examines the relationship between change in BMI over a 10-year period and knee structure as measured by MRI. It has been suggested that change in knee structure may be more likely to occur in those with early OA. Although radiographs were not available to identify participants with early signs of OA, MRI has been shown to have greater sensitivity in the detection of osteophytes [29]. Thus by excluding those with osteophytes, we have excluded those with very early subclinical OA, suggesting that our results were not due to changes in those with early preclinical and pre-radiographic OA. Another major strength of this study is that participants were asymptomatic. Thus it is unlikely that the knee changes caused weight gain. Whilst this study was able to examine the relationship between weight gain and knee structure, our power to examine the relationship between change in BMI and knee structure was more limited, since few subjects lost weight, and the age of weight gain over the 10 years of the study may have varied within the group. Given the current mean BMI of the sample was 1.4 kg less than subjects that were not included (*P *< 0.001); we speculate that the magnitude of observed association between BMI and change in BMI on knee structure may have been underestimated. Upon exclusion of subjects with osteophytes, we may have been underpowered to demonstrate an association between BMLs and BMI.

## Conclusions

This study suggests that increasing obesity in young adults, without evidence of clinical or radiographic knee OA, is associated with a detrimental effect on bone with increased prevalence of BMLs, and a non-significant increase in the prevalence of cartilage defects, which may be partially related to the presence of BML. Changes in both BMLs and defects have been previously shown to precede increased cartilage loss. Furthermore, these findings of association between BMI and BMLs and defects persisted in our population that had no radiographic OA. It is unknown whether the avoidance of weight gain in early adulthood may reduce these structural changes and diminish the risk of knee OA. Whilst the impact of weight gain at different stages of life on knee structure warrants further investigation, so too does the impact of weight loss, to determine whether this is able to reverse these structural changes.

## Abbreviations

BMI: body mass index; BMLs: bone marrow lesions; BSD: Barwon Statistical Division; CV: coefficient of variation; GOS: Geelong Osteoporosis Study; ICC: intraclass correlation coefficient; MRI: Magnetic Resonance Imaging; OA: osteoarthritis.

## Competing interests

The authors declare that they have no competing interests.

## Authors' contributions

SLB, FMC and AEW conceived and designed the study. SLB, MJH, JAP, MAK, GCN and AEW had the major role in analysis and interpretation of the data and in drafting the report. MJH, JAP, YW, and AEW supervised the statistical analysis. SLB and YW undertook measurement of knee structures. All authors contributed to drafting the report, and interpretation of the data. All authors had full access to all of the data (including statistical reports and tables) in the study and can take responsibility for the integrity of the data and the accuracy of the data analysis.
